# High-aspect-ratio nanoimprint process chains

**DOI:** 10.1038/micronano.2017.17

**Published:** 2017-07-17

**Authors:** Víctor J. Cadarso, Nachiappan Chidambaram, Loïc Jacot-Descombes, Helmut Schift

**Affiliations:** 1 Laboratory for Micro- and Nanotechnology, Research Division Synchrotron Radiation and Nanotechnology, Paul Scherrer Institut, Villigen 5232, Switzerland; 2 Micro Resist Technology GmbH, Köpenicker Straße 325, Berlin 12555, Germany

**Keywords:** cryo-etching, direct 6 write laser lithography, high aspect ratio, moulding, nanoimprint lithography, Ormocer, photonic nanofences, 2-photon polymerization

## Abstract

Different methods capable of developing complex structures and building elements with high-aspect-ratio nanostructures combined with microstructures, which are of interest in nanophotonics, are presented. As originals for subsequent replication steps, two families of masters were developed: (i) 3.2 μm deep, 180 nm wide trenches were fabricated by silicon cryo-etching and (ii) 9.8 μm high, 350 nm wide ridges were fabricated using 2-photon polymerization direct laser writing. Both emerging technologies enable the vertical smooth sidewalls needed for a successful imprint into thin layers of polymers with aspect ratios exceeding 15. Nanoridges with high aspect ratios of up to 28 and no residual layer were produced in Ormocers using the micromoulding into capillaries (MIMIC) process with subsequent ultraviolet-curing. This work presents and balances the different fabrication routes and the subsequent generation of working tools from masters with inverted tones and the combination of hard and soft materials. This provides these techniques with a proof of concept for their compatibility with high volume manufacturing of complex micro- and nanostructures.

## Introduction

High-aspect-ratio microstructures (HARMS), that is, structures with a large height/width ratio, have found many applications^1,2^ such as X-ray gratings^3^, gas chromatography columns^4^, magnetic coils^5^, X-ray telescopes^6^, micro-gears^7^, micro-capacitors with high-aspect-ratio (HAR) cantilevers^8^, and micromechanical and micro-optical elements^9,10^. These structures exhibit aspect ratios (ARs) of over 10 and almost vertical sidewalls, making them much more challenging to develop than the structures typically used in planar technology with moderate ARs of 1 to 2. They are therefore characterized by an intermediate state between two- and three-dimensional (2D and 3D), that is, a 2D planar design that is extended into the vertical dimension and often called 2½-D Ref.(11). There are several methods to produce HAR structures on typical planar substrates, most of them relying on the anisotropy of subtractive pattern transfer of a low AR masking layer into an underlying resist or substrate, that is, by using mask based ultraviolet (UV)-photolithography (PL) with an AR up to 5, silicon etching (for example, dry etching) with an AR up to 20, and direct write laser lithography (DWL) and X-ray lithography with ARs >20 Refs.(12,13,14,15). These methods are continually being improved to produce a higher AR and smaller lateral dimensions. Furthermore, they are enlarged by new techniques that are often subsets of etching processes with different advantages and disadvantages. One of those challenges is the suitability for submicron and nanopatterning, that is, structures with lateral sizes much smaller than 1 μm and the possibility to combine micro- with nanostructures in the same material.

There are three main approaches: (1) subtractive patterning by etching deep trenches into a hard material, often silicon or diamond, using a masking layer on top (resist or hard mask) and HAR nanopores in anodic alumina (AAO) via a self-limiting etching process^16,17,18,19,20,21^, as well as polymers that have been exposed to photons or electrons through a mask or by a scanning beam and are subsequently wet developed^14, 22,23,24,25,26^. Ablation via laser is a direct subtractive method for patterning substrates^27^. (2) Additive patterning is an approach that involves building up pillars, ridges, and 3D structures, often by exposing resists that are crosslinked upon exposure and wet development (that is, rinsing the non-crosslinked resist)^28,29,30,31^. (3) Finally, moulding techniques can be used to transform a pattern, for example, to mould high ridges from deep trenches and vice versa^32,33,34,35^. Two prominent examples of this are LIGA technology (a German acronym for lithography (Li, Lithographie), electroforming (G, Galvanik), and moulding (A, Abformung)) for microstructuring and nanoimprint lithography (NIL) for nanostructuring. LIGA and NIL rely on a process chain of three basic steps consisting of the production of an original structure with the desired topography (the master), working stamp fabrication (the mould used for the replication process) and replication for high-volume manufacturing (HVM)^36^. All three of these steps are critical and have a strong influence in the final structures and their functionality for applications^37^. Hence, replication is needed for the generation of the final structures and tooling. Indeed, the generation of intermediate stamps (tooling), which are needed for converting an original into a moulding tool of a material with suitable mechanical (for example, hard into soft), optical, and chemical properties, reverses the tone of the original (‘positive’ ridges into ‘negative’ trenches or vice versa). In addition, this replication step enables multiplying an original into many working stamps, strongly extending the use of a very expensive original without damaging it. The final structures require specific geometrical parameters, which need to imprint into thin initial layer to achieve low residual layers on a hard substrate with high optical transmission and the ability for chemical functionalization. These processes have to be scalable to HVM, leading to a huge number of identical devices with low defectivity. For clarity, in this work, we define the initial etched silicon or exposed polymer structures used for the replication as masters. Often, the master is fabricated in the reversed tone with respect to the final structures (that is, the master is in a negative-tone configuration and the final structure is in a positive tone), and it can be directly used as a mould for the replication process. However, it is also common to use an initial master in the same tone as the final structures (that is, the master is also in a positive tone) and an intermediate replication is required to develop a copy with reversed tone, known as the working stamp, which is used as a mould in the replication of the final structures.

**Figure 1:**
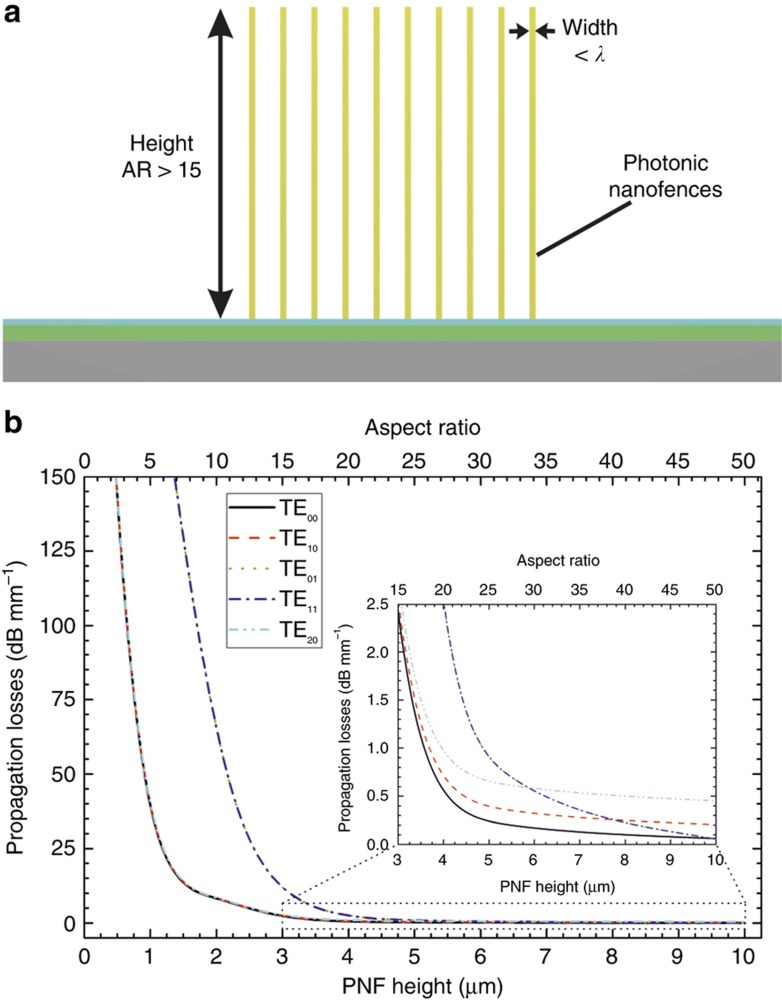
(**a**) Schematic representation of an array of photonic nanofences at a scale presenting the high aspect ratio with a width smaller than the working wavelength. (**b**) Numerical simulations of the optical losses (635 nm) of different light modes coupled into an array of photonic nanofences.

An example of nanostructures requiring an HAR for a strong optimization of their performance is the recent report demonstrating photonic nanofences (PNFs)^38^. PNFs consist of long nanoridges with lateral dimensions smaller than half of the working wavelength (that is,<300 nm) and a height in the range of a few microns (Figure 1a). These structures work as an optical waveguide capable of coupling and guiding light, but their propagation losses are directly related to their height and, thus, their AR. This relation for an array of 5 PNFs with a width of 200 nm and a 2 μm period has been determined by means of numerical simulations, as shown in Figure 1b. Optical losses increase drastically if their AR is below 15 (that is, 3 μm height). These structures have been recently developed using direct fabrication methods. However, this approach does not allow high throughput manufacturing, only prototyping of such devices and the fabrication of originals for moulding. Replication methods would be the preferred technology for the development of such structures, allowing their mass production. However, the requirements of PNFs with HAR are challenging for standard masters and replication methods^39,40,41^. This is not only because of the HAR needed for achieving low optical losses but also because of the required low residual layers of <1 μm. However, PNFs can work for a range of dimensions and configurations. Their dimensions of height and width are typically similar to those of in/out coupling optical waveguides with a core size of 5 to 20 μm. The dimensions of a single PNF are below the wavelength of the light used (that is, 200–400 nm) and the period can be varied depending on the number of PNFs. While there is a range of different solutions, for example, for guiding and sensing applications, an optimal PNF waveguide would consist of 5 to 9 ridges, that is, with periods of 5 to 0.5 μm Ref.(38), propagation distances from a few hundred μm to a few millimeters and an AR of at least 10, but concrete applications will lead to concrete geometries and distributions. In this work, three different approaches, that is, thermal nanoimprint lithography (T-NIL), ultraviolet light assisted nanoimprint lithography (UV-NIL), and micromoulding in capillaries (MIMIC), using masters fabricated with two different methods, that is, silicon etching and 2-photon polymerization (2PP) DWL, are considered, tested and compared in order to produce HAR sub-micrometric structures. Owing to manufacturing constraints and for practical reasons, in the results published here, the PNF dimensions were varied for the different manufacturing methods. In the following, we demonstrate an HAR for all processes without claiming that the limitations of the individual processes are met.

## Materials and methods

### PNF optical simulations

The simulations presented in Figure 1b were performed using the commercial software Photon Design, Oxford, UK (FIMMWAVE module) using the effective refractive index method. An array of 5 PNFs distributed in 10 μm with a refractive index (*n*) of 1.55, a width of 200 nm and different heights (*d*) was considered for this concrete example. The PNFs were simulated on top of an anti-resonant reflective substrate composed of silicon, silicon oxide (*n*=1.456, *d*=240 nm) and silicon nitride (*n*=2.001, *d*=100 nm) to enhance the coupling of light into the PNF array^38^.

### Fabrication approach considerations

The aforementioned approaches for the replication of HAR structures are based on the use of silicon etching and 2PP DWL and three different replication methods T-NIL, UV-NIL, and micromoulding in capillaries (MIMIC)^5,36^. Hard stamps, such as silicon or inorganic-organic hybrid polymers, that is, Ormocers, are ideal candidates for T-NIL with squeeze flow in which a pressure is applied for a complete filling of the structures^42^, while soft stamps, such as UV-curable polydimethyl siloxane (UV-PDMS), enable conformation to surface undulations of the moulds by capillary action^43,44,45^. First, the fabrication of the two different masters is presented.

### Silicon-based masters and moulds

**Figure 2:**
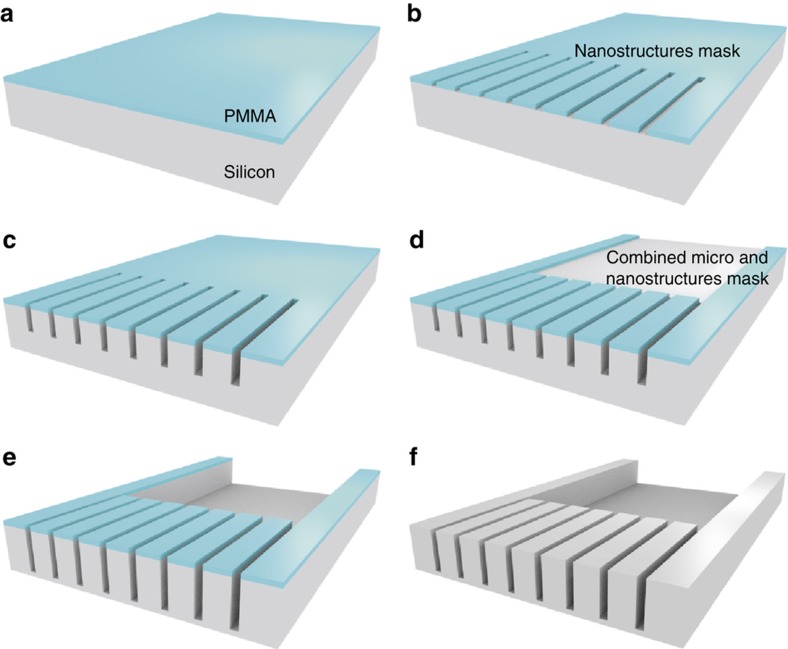
Schematic representation of a double cryo-etch process with one single PMMA layer depicted as follows: (**a**) Spin coating of the poly(methyl methacrylate) (PMMA) layer. (**b**) EBL for nanostructures. (**c**) First cryo-etch process on nanostructures. (**d**) EBL for microstructures. (**e**) Simultaneous etch of micro- and nanostructures. (**f**) Removal of the PMMA layer.

The use of dry etching is the most common approach for the fabrication of silicon moulds for NIL. However, for the development of deep structures, the preferred method is the use of the Bosch process or deep reactive ion etching, which produces trenches with vertical sidewalls through an anisotropic process based on repetitive switching between etching and sidewall passivation. This technique is extremely successful for a myriad of applications; however, it is also well known to result in the formation of high roughness in the form of scalloping in the lateral sidewalls^17^. This roughness is often irrelevant, but for the demoulding of HAR, it becomes critical because it dramatically increases demoulding forces (that is, friction), resulting in the ripping of the structures. Furthermore, it will also result in an increase in optical losses in the development of the proposed PNFs. To prevent this, silicon cryo-etch has been used with an SF_6_:O_2_ plasma^16^. This method uses low temperatures (typically below −100 °C) at which a passivating layer of SiO_*x*_F_*y*_ is formed. This enables a highly anisotropic etching in the vertical direction that depends on the SF_6_:O_2_ ratio and the temperature^46^. This is of high interest for the fabrication of masters for HAR nanostructures, including the PNF with its nanoridges along with micro-sized multimode waveguides. As with other plasma-based dry etching techniques, the etch rate has a strong dependence on the size of the structures to etch. Hence, an approach allowing compensation for this depth difference is proposed, as depicted in Figure 2. Initially, a layer of poly(methyl methacrylate) (PMMA) composed of 950 kg mol^−1^ (micro resist technology, Germany) dissolved at 5% in anisol is spin coated at 1000 rpm on the silicon wafer (Figure 2a) and pre-baked at 180 °C for 10 min. This is followed by the first electron-beam lithography (EBL) exposure step (EBPG 5000PlusES, Vistec, Jena, Germany) at 100 keV with a dose of 840 μC cm^−2^ to open the nanostructures (Figure 2b). Then, the nanostructures are etched into the silicon by a cryo-etch process (Figure 2c). At this point, the usual procedure will be to remove the remaining PMMA layer and repeat the previous steps. Instead, here the same PMMA layer is maintained for a second e-beam process (Figure 2d). This allows the opening of the micro-sized structures while maintaining the mask for the nanostructures unmodified. Then, a second cryo-etch process is performed (Figure 2e), considering the etch rate at both the micro- and nanostructures in order to obtain similar final depths. Both cryo-etch steps were performed on a Plasmalab 100 ICP tool (Oxford Instruments, Yatton, Bristol, UK) at −105 °C with a mixture of SF_6_ (49 sccm) and O_2_ (10 sccm) at a pressure of 20 mTorr and radio frequency and ICP powers of 10 and 600 W, respectively. Etching times were 50 and 60 s for the first and second etching steps, respectively^47^. Finally, the PMMA layer can be removed (Figure 2f), and the Si master is ready for use.

### 2PP DWL-based masters for the generation of working stamps

**Figure 3:**
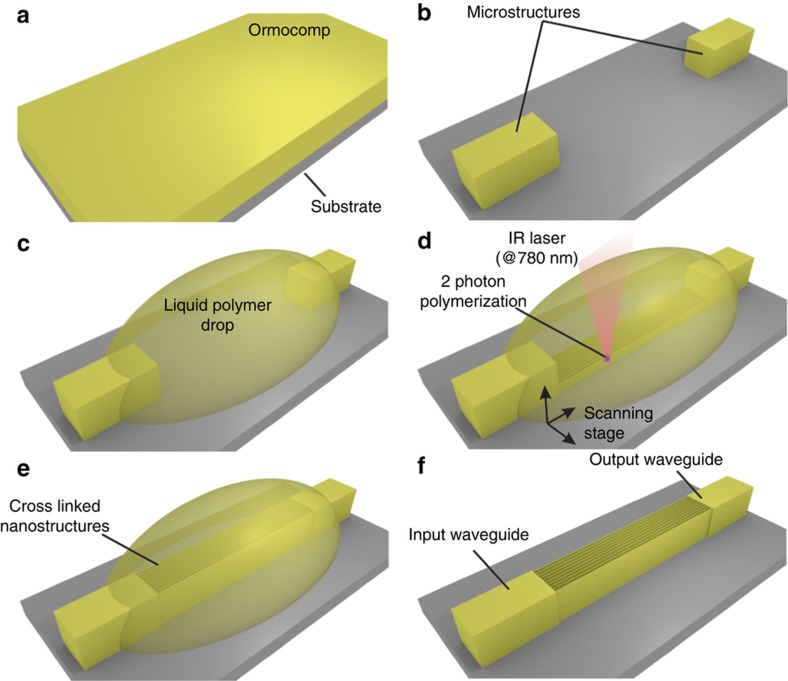
Schematic representation of a ultraviolet (UV) photolithography (PL) and 2PP direct laser write (DWL) process for master fabrication. (**a**) Spin coating of OrmoComp. (**b**) UV-PL to form micro-sized structures. (**c**) Deposition of a polymer liquid drop on the region of interest. (**d**) DWL by 2PP to from the (**e**) nanostructures. (**f**) Wet development of the nanostructures to remove non-crosslinked material.

This approach allows the fabrication of complex moulds with 3D shapes and, particularly, HAR structures with sub-μm lateral size and vertical sidewalls. The 2-photon polymerization (2PP) direct write laser (DWL), as an additive technique, allows the sequential build-up of structures by scanning the focus of a laser beam through a resist, thus enabling crosslinking of only the volume of the resist (known as a voxel) at the centre of its focal point where the energy density is above certain threshold and where the simultaneous absorption of 2 photons of lower energy occurs (that is, crosslinking a UV-sensitive resist using a laser in the near infrared at 780 nm). The master structures were directly written from the design file using a commercial ‘Photonic Professional GT’ tool from Nanoscribe GmbH, Germany. To combine both micro- and nanostructures, as the ones used for PNFs, DWL was used in combination with standard UV photolithography to develop polymer moulds as an alternative to the Si etched ones, as depicted in Figure 3. This process results in the fabrication of “positive” hard polymeric structures, that is, an Ormocers or IP-Dip (a negative-tone photoresist optimized for high resolution in 2PP processes, Nanoscribe). OrmoComp (micro resist technology, Eggenstein-Leopoldshafen, Germany) was spin coated on a silicon wafer at 4000 rpm to produce a 10 μm thick layer. Conversely, OrmoComp diluted in propylene glycol methyl ether acetate (PGMEA, 1:2, w/w) was spin coated at 1200 rpm to produce a 4 μm thick layer. The diluted OrmoComp was pre-baked at 95 °C for 5 min. The OrmoComp was exposed on proximity in a mask aligner (MA6, Suss MicroTec, Munich, Germany) with a dose of 1500 and 800 mJ cm^−2^ for the 10 and 4 μm layers, respectively. After development in PGMEA for 10 min and rinsing in isopropanol to obtain the final microstructures, a drop of OrmoComp or IP-DIP was deposited in the areas were the nanostructures were added. The wafer was then placed on the DWL tool, and the nanostructures were exposed through the liquid resist using 2PP at 780 nm (absorption at 390 nm). Finally, the structures were developed once more in PGMEA for 10 min and rinsed in isopropanol. To prevent the HAR nanostructures from collapsing, the isopropanol was removed by performing a supercritical drying process.

### Mould copies replicated from 2PP DWL masters

To obtain intermediate working stamps with a negative tone, a first replication of such structures is needed. This was done with UV-PDMS (X-34–4184, Shin-Etsu, Japan, distributed by micro resist technology, as KER-4690). The fabrication of the working stamp was achieved by depositing a drop of the polymer on the 2PP master and carefully placing a glass substrate on top. Once the polymer was covering the whole area of the master, it was exposed to UV light for crosslinking and mechanically released. The final thickness was in the range of 0.5 mm, and the process parameters are described below (UV-NIL).

### Antisticking layer coating

All masters and stamps were coated with an antisticking layer, using a homemade vacuum chamber (4 liters) for molecular vapor deposition (MVD) of perfluorinated silanes. First, to avoid damage by excessive RIE treatment, the moulds were exposed to a gentle low power oxygen plasma (Oxford Plasmalab 80, 20 W, 30 s) prior to coating. Immediately after, they were placed in a vacuum chamber at 100 Pa and 5 μL was injected into the chamber, by which the silanes evaporated and precipitated on the moulds. After 10 min, the excess silane in the chamber was removed and the silane monolayer coated moulds were ready to be used. As silanes, we used a 1:1 mix of undiluted F_13_-OTCS (tridecafluoro-1,1,2,2-tetrahydrooctyl)-trichlorosilane (SIT 8174) and F_13_-OMCS (tridecafluoro-1,1,2,2-tetrahydrooctyl)-dimethylchlorosilane (SIT 8170) from ABCR, Karlsruhe, Germany. The co-deposition of trichloro- and monochlorosilanes is known to lead to surfaces with decreased bulk polymerization between the unbound silane molecules in comparison to pure trichlorosilane, but they provide the same water contact angle (117°) on the surface^48^.

### Nanoimprint lithography

Si moulds were initially prepared for thermal NIL (T-NIL) with thin layers of PMMA, which is the classical process for achieving low residual layers in thermoplastic polymer resists^39,42,49^. However, thermal processes with non-crosslinked materials impose high stress on the material and often result in ripping during demoulding. For this reason, alternative methods and materials were considered, that is, UV-NIL and MIMIC. In contrast to the generation of thick working stamps, a special interest lies in obtaining thin residual layers for which the initial layer thickness has to be minimized.

**Figure 4:**
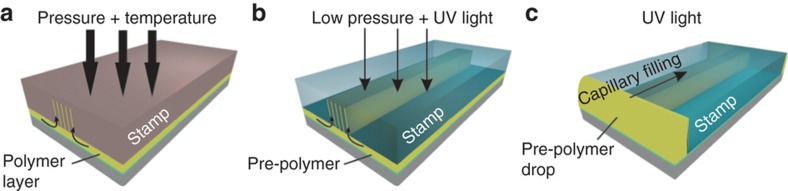
Schematic representation of the three applied replication processes: (**a**) T-NIL, in which a hard stamp is used to press a heated thermoplastic polymer film and fill the cavities of the stamp by squeeze flow and capillary forces. (**b**) UV-NIL, in which a transparent stamp is placed upon a liquid prepolymer layer to fill the cavities by capillary action, and subsequent UV light exposure is used to crosslink the polymer. (**c**) In micromoulding into capillaries (MIMIC) a transparent stamp with an open, continuous fluidic network is placed on the substrate and filled by capillary action from the side with a liquid prepolymer that is crosslinked by ultraviolet (UV) light exposure.

T-NIL (also known as hot embossing lithography), depicted in Figure 4a, is based on the use of a thermoplastic material that is heated above the glass transition temperature to make it viscous enough so that it can be deformed and moulded into the structures of the stamp by applying relatively high pressure. Thermal imprint needs to squeeze the polymer below the stamp protrusions into the mould cavities (that is, the trenches in the stamp). At short times or low temperatures, the ability of the PMMA to flow is very limited; therefore, hard stamps (Si or OrmoStamp) and high pressures are usually required. Processing at long times or higher temperatures can enhance the ability of the PMMA to flow. However, according to the wetting behavior, this can be compromised by the ability of the viscous material to flow along the ridges by capillary forces. Demoulding is generally enabled owing to low friction and rigid materials with high cohesion and good adhesion to the substrate. Antisticking layers (ASL) generally reduce demoulding forces but are detrimental to the filling of narrow stamp structures owing to reduced wetting^40,41^. T-NIL was used to produce a PMMA film on a glass substrate using the HEX 03 press from Jenoptik Mikrotechnik, Jena, Germany. PMMA pellets with 120 kg mol^−1^ (micro resist technology, Germany) were dissolved in anisole to a desired concentration so that, after spin coating at 2000 rpm followed by soft-bake at 150 °C, a 2.5 μm thick film was obtained. The imprint was performed at a pressure of 10 MPa and an imprint temperature of 180 °C for 30 min. Typically, vacuum (~500 Pa) is applied prior to imprint, and after cooling, manual demoulding was performed at approximately 40 °C. To avoid excess lateral flow, which does not allow homogeneous filling of the trenches, the pressure was reduced to 2.5 MPa.

A room temperature alternative to T-NIL is UV-NIL (Figure 4b). In this process, instead of a thermoplastic, a liquid UV-sensitive prepolymer is spin coated or deposited on the substrate, and the stamp is pressed against it to fill the cavities. Then, the prepolymer is exposed to UV light through the substrate or through the stamp (one of them has to be transparent to UV light, that is, glass or PDMS) to crosslink it, obtaining the final polymer structures. UV exposure was performed with an ELC-500 from the Electro-Lite Corporation, Bethel CT, USA with a peak intensity at 365 nm. The exposure doses were 18, 7.2, and 5.4 J cm^−2^ for IP-Dip, OrmoStamp/OrmoComp, and UV-PDMS, respectively. All of these are mildly overexposed compared to the manufacturer’s recommendation. All of the stamps were coated with ASL (perfluorinated silanes) as described above.

As an alternative to standard imprinting methods, MIMIC was tested to prevent the formation of the residual layer^50^. In this method, the mould is cut open on both ends and directly placed on the substrate, forming a microfluidic network. When using elastomeric materials for the mould (that is, UV-PDMS), conformal contact between the mould and substrate can be achieved. This is monitored using an optical microscope by looking for the interface between the mould and the substrate. Then, a drop of the liquid prepolymer (that is, OrmoComp or OrmoClear) is placed on one end of the mould in contact with one side of the open channel, and the stack is placed in a vacuum chamber at 100 Pa for 10 min in order to facilitate the filling of the HAR nanostructures. By pulling vacuum, the channels are evacuated, and capillary action is facilitated, enabling filling within a few minutes. Since it is known that the PDMS elastomer is permeable to gases^51,52^, the air is also able to escape by diffusion through the elastomer. After filling, the Ormocer was exposed through the stamp or the substrate in UV light with a dose of 7.2 J cm^−2^ (Figure 4c). After hardening, the stamp was peeled from the surface and the micro- and nanostructures were demoulded. Previous works have demonstrated MIMIC-like process to fabricate AR 1 structures in OrmoCore^53^, but the aim was to achieve an AR>10 in this work, as needed for PNFs.

The requirements are more delicate for HAR structures because thin ridges in the mould could bend and collapse during contact or capillary action. Furthermore, it is expected that sparse trench structures with larger periods (and thus larger intermediate segments) are mechanically more stable and enable a better sealing towards the substrate between the individual trenches. Therefore, for testing MIMIC, we chose PNFs with 1 to 3 ridges only. We also used undiluted polymers with quite high viscosity (2 Pa s), which is known to slow down the filling significantly. However, for accelerating the process and for complete filling, we chose vacuum instead. We conclude that vacuum can help to fill structures faster and is particularly advantageous for mixed micro- and nanostructures where air voids could be eliminated.

## Results

The replication of HAR structures combining micro- and nano-features, such as PNF, presents challenges both in mould copying and final replication. For this reason, two different technologies (cryo-etch and 2PP DWL) and three different replication technologies were tested (thermal and UV-assisted NIL and MIMIC) to produce the masters, replicate working stamps and imprint the final structures. The methods are different in AR but are all suitable for the fabrication of HAR structures, however, with different yields. The successful results are summarized in Table 1.

### Cryo-etch

**Figure 5:**
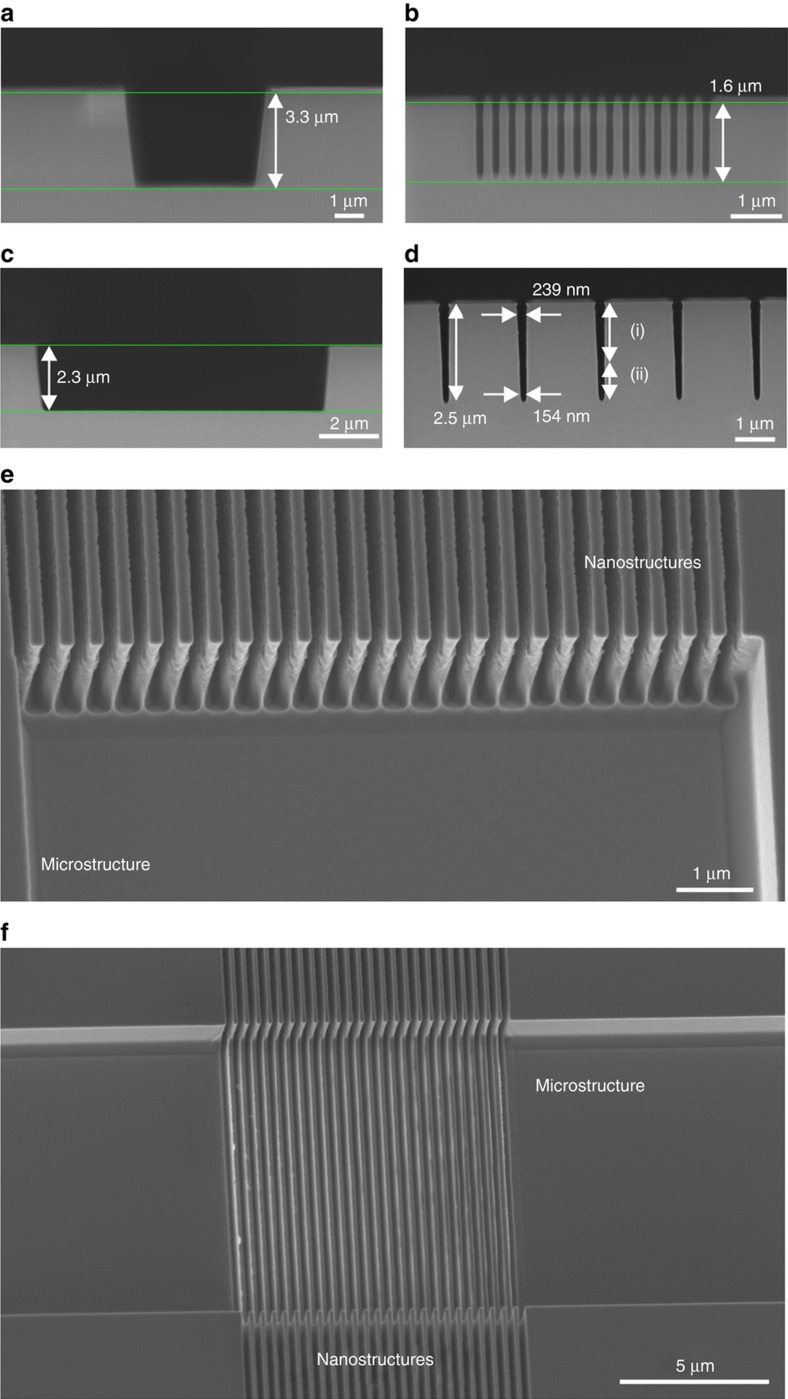
Scanning electron microscope images of silicon moulds fabricated using the cryo-etch process. Standard processing of (**a**) micro- and (**b**) nanosized trenches with a distance between nanostructures of 330 nm and the resulting difference etch rates. Double etching process on (**c**) micro and (**d**) nanosized structures (*d*=2 μm) resulting in trenches with equivalent depths. (**e**) Junction between micro- and nanostructures (pitch 400 nm). (**f**) Hierarchical structures featuring nanochannels (*d*=400 nm) etched next to and at the bottom of a microchannel.

The cryo-etch technique allows deep etching of silicon with control on the sidewall inclination and no scalloping. As the scanning electron microscope micrographs (SEM, Supra VP55, Zeiss, Oberkochen, Germany) show, by comparing Figure 5a for a 5 μm wide aperture and Figure 5b for an array of 200 nm wide nanostructures, the former resulting in an etch depth of 3.3 μm and the latter in only 1.6 μm after 1 min of etching. By using the method described in Figure 2, it was possible to develop combined micro- and nanostructures with equivalent depths, as shown in Figures 5c and d. Positive slope sidewalls were obtained in order to facilitate the later demoulding of the structures. A sidewall angle of 88.1°±0.6° was obtained by controlling the process parameters (mainly temperature and SF_6_:O_2_ ratio). Defects were not observed at the boundary between the two etching steps at the nanostructures or in the transition between the micro- and nanostructures (Figure 5e). This technique was also tested to overlap both micro- and nanostructures to obtain hierarchical structures, as shown in Figure 5f.

### Photolithography, 2PP DWL, and working stamp fabrication

**Figure 6:**
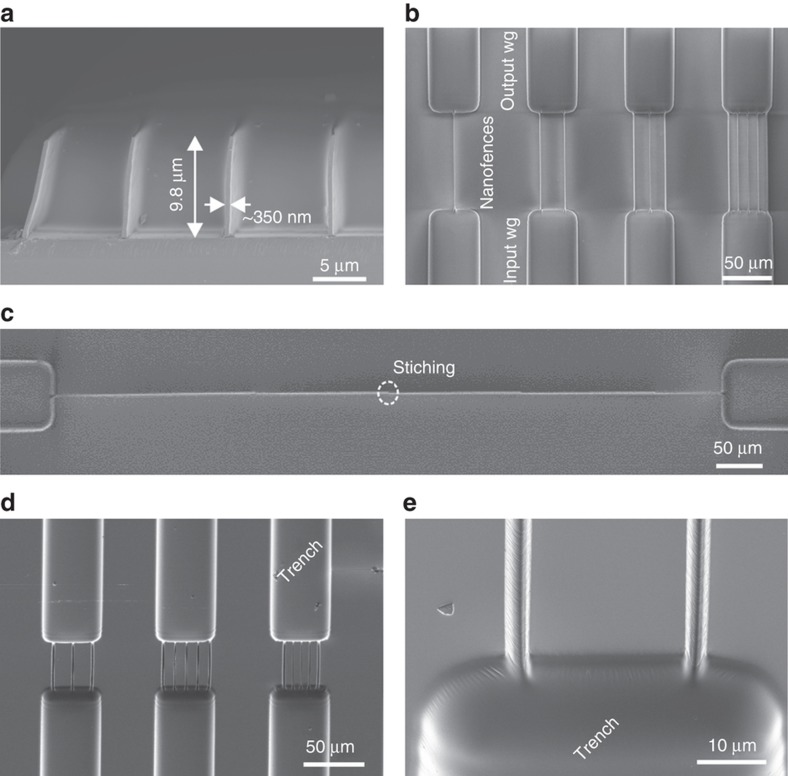
SEM images of the fabricated masters using the 2PP method. (**a**) HAR nanostructures (an AR of 28, *d*=5 μm) obtained by 2-photon polymerization (2PP)-direct laser write (DWL). (**b**) Combination of micro and nanosized structures featuring photonic nanofences (*d*=n.a., 25, 16.7, and 10 μm). (**c**) Long nanostructure (500 μm) showing the write field limits and a slight stitching error. (**d**) Replicated (trench) structure from the 2PP master into UV-curable polydimethyl siloxane (*d*=16.7, 10, and 5 μm) and (**e**) a larger magnification image showing the transition between micro- and nanostructures.

Using 2PP DWL, very HAR structures with an AR up to 28, as shown in Figure 6a, were developed. The nanostructures were aligned between the microstructures, and it was possible to obtain different arrays in multiple configurations (Figure 6b). The fabricated nanostructures exhibited vertical sidewalls (90°±0.01°). Writing fields with this selected technique are limited to a circle with a diameter of ~250 μm. Hence, the fabrication of longer nanostructures rendered some stitching. However, this can be calibrated and minimized, as shown in Figure 6c with a stitching error of ~50 nm. These structures were fabricated in a positive configuration; hence, in order to obtain moulds, the masters needed to be copied into another material. Two different materials were tested: OrmoStamp and UV-curable UV-PDMS. In both cases the selected prepolymer was cast on the 2PP fabricated master and cured under UV light. The released moulds presented the required negative features for further replication of the HAR nanostructures. Figure 6d shows an example of the obtained moulds with both micro- and nanostructures replicated in the PDMS (trenches). The smooth transition between micro- and nanostructures can be seen in Figure 6e.

### T- and UV-NIL

**Figure 7:**
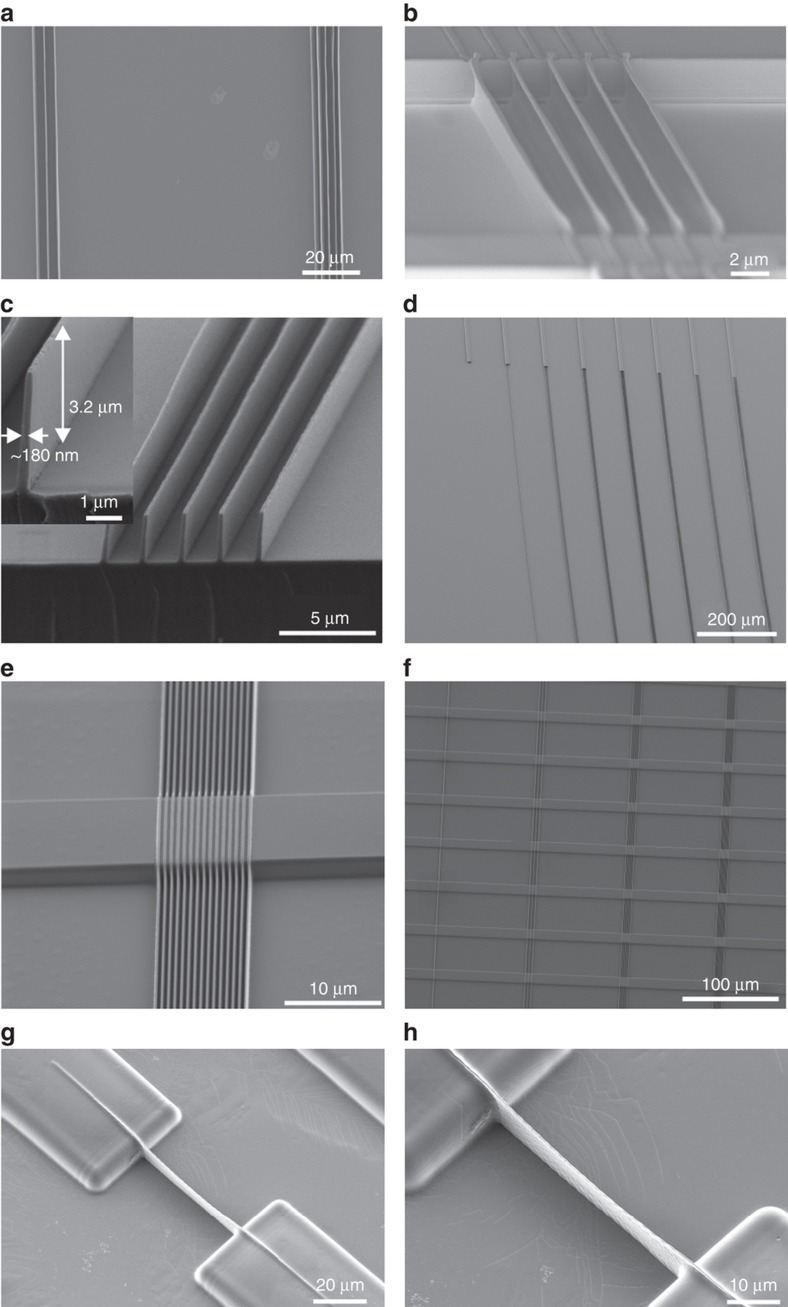
Nanoimprinted photonic nanofences obtained from different materials and moulds (details in Table 1). T-NIL replicated (**a**) and (**b**) long (up to several millimeters) and high aspect ratio (up to 17.5) nanostructures in poly(methyl methacrylate) (PMMA). UV-NIL replicated structures in OrmoComp with an HAR of 17 (**c**) and 1 mm long structures (**d**) from a cryo-etched Si stamp. (**e**) and (**f**) Hierarchical structures replicated from the same stamps on a large area. (**g**) and (**h**) UV-NIL replicated structures with an HAR of 20 in OrmoComp obtained from a UV-PDMS mould copied from a 2-photon polymerization (2PP)-direct laser write (DWL) master.

Although it was possible to obtain long structures and structures with an HAR of 17 (Figures 7a and b) using T-NIL, the yield was extremely low (<5%), and most of the structures were broken or detached from the substrate. Furthermore, the residual layer was not yet optimized for the PNF device. UV-NIL using OrmoComp and OrmoStamp on glass substrates rendered a much higher yield (~70%) with an HAR of 17.5, as shown in Figure 7c. The nanostructures had widths of ~180 nm and heights up to 3.2 μm. It was possible to obtain extended nanoridges with lengths of several millimeters (>5 mm, Figure 7d). The moulds containing hierarchical structures were also tested, as presented in Figure 7e. Nanostructures both on top of the microstructures and on the substrate were well defined. This replication was possible on relatively large areas, as Figure 7f shows. However, UV-NIL profits from the use of soft stamps since it allows low pressure imprint into liquid precursors owing to their low viscosity and allows trapped air to escape by diffusion through the elastomer. For process sequences involving more than one replication step, a good balance between hard and soft properties of stamps and the moulding material is needed, along with a good choice of pressure and the ability to achieve high resolution with soft stamps (UV-NIL). Soft stamps may also be used in T-NIL^45^, but the stamp becomes compressed and, owing to lateral expansion, narrow trenches are easily clogged. The use of the PDMS moulds fabricated from the 2PP DWL masters in UV-NIL allowed the formation of nanostructures up to 9.8 μm height with widths down to 350 nm, resulting in an HAR of 28, as shown in Figure 7g. Nanostructures with widths of only ~200 nm were also obtained, with a maximum height of 4 μm, resulting in an HAR of ~20.

The main disadvantage of both imprinting methods was the formation of a large residual layer ~10 μm thick. Complete filling was only possible if the initial layer was thick enough to provide enough material to flow into the cavities. Although this residual layer will not be a problem for some applications, for the correct optical behavior of PNFs, it needs to be reduced at least by a factor 10. Thinner initial layers were not possible because of the low density of trenches; thus, the restrictions involved in squeeze flow as well as capillary effects involving lateral flow caused clogging at the PNF interfaces and depletion within the trenches^54^.

### MIMIC

**Figure 8:**
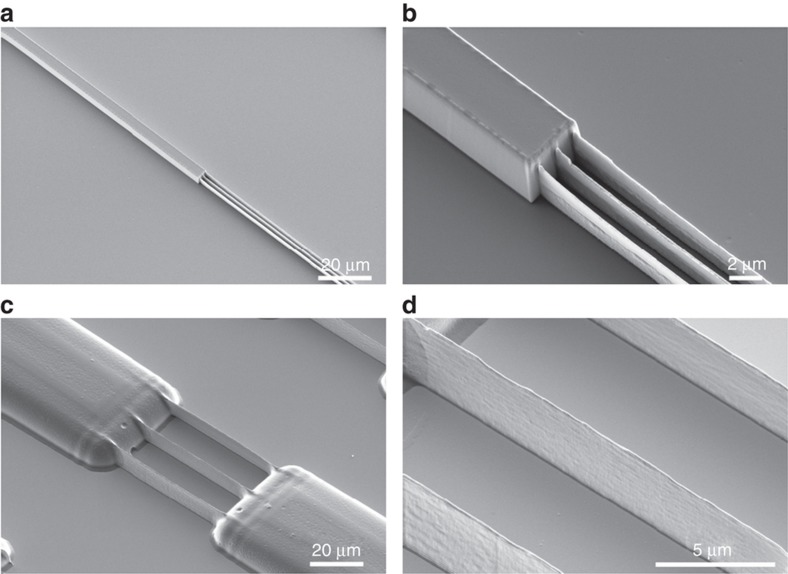
Ultraviolet-micromoulding into capillaries replicated PNFs in OrmoComp using (**a**) and (**b**) a cryo-etch silicon mould and (**c**) and (**d**) a UV-curable polydimethyl siloxane mould copied from a 2-photon polymerization (2PP)-direct laser write (DWL) master. Owing to the sealing between mould and substrate, the residual layer in **c** and **d** is negligible.

When in contact with one side of the open channels, the sample was allowed to rest. The inclusion of the HAR nanostructures between the microstructures prevented the complete filling of the long structures by capillary forces. For this reason, after depositing the prepolymer drop, the moulds were placed under vacuum to assist the filling of the nanochannels. Figure 8 shows the replicated structures with OrmoComp based on the use of a hard Si mould (Figures 8a and b) and a soft UV-PDMS 2PP mould (Figures 8c and d). For the sample obtained from the Si mould, a residual layer of ~1 μm was obtained, which is still too high. Conversely, the sample developed from the PDMS mould exhibited no residual layer. This difference is because the PDMS mould is able to conform to surface undulations and adhere to the substrate prior to the prepolymer deposition, preventing it from flowing in between the protrusions and the substrate. The obtained HAR values were 17 for the Si mould and 28 for the PDMS mould, rendering equivalent results to the ones obtained by NIL.

## Discussion

The use of cryo-etching for the formation of an HAR silicon mould has a number of advantages. It was possible to combine both micro- and nanostructures by double exposing a single layer of PMMA, resulting in a variation of only 8% in height. Furthermore, this method proved to be extremely versatile, allowing the formation of hierarchical micro- and nanostructures. No defects could be observed either in the transition between the two etching steps at the nanostructures or in the transition between the micro- and nanostructures. By controlling the process parameters, it was possible to produce the master structures with slightly positive slopes in order to facilitate demoulding of the replicated structures. This, combined with an ASL, allowed a maximum AR of 17 for a nanostructure 180 nm wide and 3.2 μm high. The resolution was limited by the fabrication methods of the master, the combination of EBL and the etching process, while the height was limited by the demoulding of the structures. Higher structures broke during demoulding, preventing their successful replication. With this HAR, it was only possible to use the mould between 10 and 20 times before it had to be cleaned and new ASL applied. The use of thermoplastic polymers was tested with T-NIL using PMMA, but the yield was very low (<5%) and most of the nanostructures broke during demoulding. This is not only because of the brittleness of the material but also because of stress induced by the thermal contraction during the cooling in the thermocycle. Furthermore, thermoplastic PMMA is not crosslinked and thus is mechanically less robust. Other thermoplastic polymers were considered but not tested. The mechanical properties of crosslinked materials processed at room temperature, here, the UV-curable Ormocers (that is, OrmoStamp, OrmoComp, or OrmoClear), were more appropriate for the successful replication of these HAR structures. Consequently, these materials were replicated by UV-NIL. They mostly differ in viscosity before crosslinking and have slightly different mechanical and optical properties. The results were identical in terms of resolution and AR to the ones obtained with PMMA but with a larger rate of success in the processing. One reason for this is the higher cohesion of crosslinked materials, including the process of shrinkage due to crosslinking, which enables the ridges to shrink away from the surrounding cavities. This was further enhanced by performing the demoulding at reduced temperatures, as previously described^55^. This process was validated not only with the HAR PNFs but also by replicating large areas of hierarchical structures combining both micro- and nanopatterns.

It has been previously demonstrated that the use of 2PP DWL allows the formation structures with extreme HAR values of up to 45 Ref.(38). Hence, such structures were tested as masters for the development of working stamps that could yield to HAR replication processes. Furthermore, the combination of UV-PL for the development of microstructures with the 2PP DWL for the nanostructures proved to be a straightforward method to combine them. These working stamps were replicated into UV-PDMS, which allowed the replication of trenches with an AR up to 28, with structures close to 10 μm in height and 350 nm in width. Despite the reduction in resolution (from 180 to 350 nm), this is a significant improvement in absolute HAR compared to the etched Si masters. Demoulding of the soft PDMS from the hard polymer nanoridges of the 2PP DLW stamp involved smaller detachment forces than the OrmoStamp copies from silicon trenches. The UV-PDMS has a higher tensile strength when compared to conventional thermocurable PDMS Sylgard 184 from Dow Corning, Midland MI, USA (7.7 versus 3.5 MPa)^45^, which is smaller than those for the hard acrylic materials, such as IP-Dip (50–70 MPa) and PMMA (90 MPa). Ormocers are also considered as hard materials but are less brittle than IP-Dip and PMMA and are not elastic, similar to PDMS. Hence, we conclude that the combination of these materials with the proposed technologies allowed this improvement in the fabrication of the HAR nanostructures. It was possible to achieve a resolution close to the one obtained in the Si masters (200 nm in IP-Dip vs 180 nm in silicon) but at expenses of reducing the total height of the nanostructures to 4 μm. Still, this resulted in an improved AR of 20, which was closer to the AR of 18 obtained with the Si moulds. These results may indicate that the AR not only depends on the manufacturing process but also depends on a constant directly related to the absolute dimensions and geometry of the fabricated structures.

An additional advantage of the 2PP DWL approach is the possibility to use the original DWL master multiple times to generate the PDMS moulds, which in turn can also be used several times before they degrade. The main disadvantages are the higher roughness of the sidewalls (which can be observed when comparing the different SEM images in Figure 7 (that is, Figures 7c and h)) and the stitching error present for nanostructures >250 μm. The roughness in the ridges is mostly due to the limited size of the scanned voxel in which 2PP occurs and the limited accuracy of the stage of the DWL tool. The PDMS moulds allowed the replication of the final structures into Ormocers, similar to what was observed in the Si moulds, but with the expected improvement of HAR. Once more, ASL was required for NIL in order to achieve demoulding, although, in this case, it was not required to cool down the structures prior to demoulding.

All described NIL processes, with both Si and PDMS moulds, resulted in the formation of large residual layers (that is, 10 μm for both T-NIL and UV-NIL). Reduction of the residual layer in NIL is a major field that presents a number of challenges, especially if HAR structures are being processed. Thus, in order to overcome this issue, MIMIC was shown to be a viable alternative using both Si and PDMS moulds. In MIMIC the mould is already in intimate contact with the substrate, whereas in NIL, the stamp has to squeeze the polymer sideways from below the stamp protrusions. At the same time, the filling of the trenches needs considerable flow of material, which can only be provided by squeeze flow or capillary action. In addition, the filling of both the Si and PDMS moulds in MIMIC by capillary action alone was not possible because of the large viscosity and low surface energy of PDMS opposing the wetting of the HAR nanostructures. However, placing the samples in vacuum during the filling allowed the completion of this filling, achieving both the micro- and nanostructures replication. Once more, Ormocers were the materials of choice for the replication in this process. In addition to the resolution and height differences described above and directly related with the used moulds, the main difference in this method between the Si and the PDMS moulds was the formation of the residual layer. While the flexible PDMS mould adhered to the substrate over all its protrusions, this was not the case for the rigid Si mould. This resulted in a relatively thin layer of prepolymer that flowed between the Si mould and the substrate, forming a 1 μm thick residual layer. Conversely, the polymer did not spread to the regions of the surface contacted by the PDMS mould, achieving simultaneously an HAR (up to 28), no residual layer, as expected, and structures not only complying to but also exceeding the requirements for the development of PNFs. In the future, particularly in view of upscaling replication to large scale roll-to-roll processes, a combination of MIMIC and NIL could help to achieve low residual layer imprinting of thermoplastic and UV-curable materials. For this, resist transfer methods such as liquid transfer imprint lithography (LTIL) could be advantageous^56,57^.

## Conclusion

Different methods were successfully tested to obtain master originals, working stamps and replicas with HARs. The PNFs were a particularly useful structure for the demonstration of the challenges of mixed micro- and nanostructures. For flexibility with future applications, the choice between subtractive patterning by cryo-etching and additive patterning by 2PP DWL is particularly advantageous when combined with tone-reversal by replication techniques. The combination of micro- and nanostructures in the masters required two novel approaches: (i) a double exposure process of PMMA, in which the same mask is used twice in two successive EBL and cryo-etching steps, allowing the straightforward integration of micro- and nanostructures and the formation of hierarchical structures; and (ii) a hybrid approach using scanning lithography (2PP DWL) only for the nanostructures, while microstructures were patterned using standard PL, which allows higher throughput and the fabrication of large area stamps (100 mm wafer sizes) in a cost-efficient way. Copying is particularly successful if soft stamps are replicated from hard masters and are used to pattern the functional structures. Here MIMIC with UV-curable Ormocers was the only viable route to achieve an HAR of up to 28 for submicron structures with no residual layer. Although MIMIC is still a process used in a laboratory environment, it could open the way for HVM polymeric integrated photonic devices on hard substrates through replication. The main challenge, however, lies in the right combination of methods and materials, and, in the future, in the design of complex geometries for both optimized function and HVM with high yields.

## Figures and Tables

**Table 1:** Summary of the results obtained using the different moulds and replication techniques

Master	Mould	Process	Replicated materials	Max. AR (*h*:*w*)	Resolution at max. AR (nm)	Resolution at max. AR (nm)	Max. resolution/corresponding AR (nm)/(*h*:*w*)	Residual layer (μm)
Silicon cryo-etch (negative)	Si (master)	T-NIL	PMMA	17	180	180	180/17	~2
	Si (master)	UV-NIL	OrmoStamp OrmoComp OrmoClear	17	180	180	180/17	>10
	Si (master)	UV-MIMIC	OrmoComp	17	180	180	180/17	~1
OrmoComp 2PP DWL (positive)	UV-PDMS, OrmoStamp	UV-NIL	OrmoComp OrmoClear	28	350	350	200/20	>10
	UV-PDMS	UV-MIMIC	OrmoComp	28	350	350	200/20	~0

Abbreviations: AR, aspect ratio; UV-MIMIC, Ultraviolet-micromoulding into capillaries; UV-PDMS, UV-curable polydimethyl siloxane; 2PP DWL, 2-photon polymerization-direct laser write.
